# Dynamics and frequency of Gag transmitted polymorphisms in Zambia

**DOI:** 10.1186/1742-4690-9-S2-P156

**Published:** 2012-09-13

**Authors:** M Schaefer, J Carlson, D Claiborne, J Prince, W Kilembe, J Tang, P Farmer, R Shapiro, T Ndung'u, J Frater, P Goulder, R Kaslow, S Allen, P Goepfert, D Heckerman, E Hunter

**Affiliations:** 1 Emory University, Atlanta, GA, USA; 2 Microsoft Research, Redmond, USA; 3 Zambia Emory HIV Research Project, Lusaka, Zambia; 4 University of Alabama-Birmingham, Birmingham, USA; 5 Harvard School of Public Health, Boston, USA; 6 University of KwaZulu-Natal, Durban, South Africa; 7 Oxford University, UK; 8 Unviersity of Alabama-Birmingham, USA

## Background

HIV immune escape is not random and follows a predictable mutational path in response to the HLA alleles carried by an individual.

## Methods

Using 143 epidemiologically linked transmission pairs from a Zambian cohort we assessed: (1) the frequency of Gag polymorphisms circulating in the population, (2) if the polymorphisms could be associated with the infected individual’s HLA alleles, (3) the frequency at which polymorphisms are transmitted, and (4) the relevance of the transmitted polymorphisms (TP) to the newly infected individual’s HLA-I alleles.

## Results

We observed a median of 35 (range 23-66) polymorphisms per chronically infected individual in Gag and 42% of these polymorphisms could be associated with the individual’s HLA (16% statistically linked, 26% epitope analysis). When transmission of these polymorphisms was assessed, we observed that the majority of these polymorphisms (84%) are transmitted to the epidemiologically linked partner and, of these TP, an equivalent fraction (43%; 11% statistically linked, 32% epitope analysis) were relevant to the newly infected individual’s HLA-I alleles. In 81 transmission pairs observed during the first two years of infection, we observed a very low overall reversion rate (4%/year) of the TP. Reversion was not uniform and when p17, p24, p2, p7, p1, and p6 are examined individually, TP in p17 and p2 exhibited the highest frequency (2.5x) of reversion events based on amino acid length. Four CTL-targeted positions were identified where a majority of TP reverted to consensus over the two years of follow up, consistent with a reduction in fitness following transmission.

## Conclusion

These data indicate: (1) HLA-I associated polymorphisms are stable and circulating frequently in the Zambian population, (2) individuals are acquiring HIV-I variants with a high frequency of polymorphisms relevant to their HLA-I alleles, and (3) reversion is slow and not evenly distributed across Gag.

